# Vital Signs: Foodborne Norovirus Outbreaks — United States, 2009–2012

**Published:** 2014-06-06

**Authors:** Aron J. Hall, Mary E. Wikswo, Kimberly Pringle, L. Hannah Gould, Umesh D. Parashar

**Affiliations:** 1 Division of Viral Diseases, National Center for Immunization and Respiratory Diseases, CDC; 2 EIS officer, CDC; 3 Division of Foodborne, Waterborne, and Environmental Diseases, National Center for Emerging and Zoonotic Diseases, CDC

## Abstract

**Introduction:**

Norovirus is the leading cause of acute gastroenteritis and foodborne disease in the United States, causing an estimated one in 15 U.S. residents to become ill each year as well as 56,000–71,000 hospitalizations and 570–800 deaths, predominantly among young children and the elderly. Whereas noroviruses often spread through person-to-person contact, foodborne transmission can cause widespread exposures and presents important prevention opportunities.

**Methods:**

CDC analyzed 2009–2012 data on suspected and confirmed norovirus outbreaks reported by state, local, and territorial health departments through the National Outbreak Reporting System (NORS) to characterize the epidemiology of foodborne norovirus outbreaks.

**Results:**

During 2009–2012, a total of 1,008 foodborne norovirus outbreaks were reported to NORS, constituting 48% of all foodborne outbreaks with a single known cause. Outbreaks were reported by 43 states and occurred year round. Restaurants were the most common setting (64%) of food preparation reported in outbreaks. Of 520 outbreaks with factors contributing to contamination reported, food workers were implicated as the source in 70%. Of 324 outbreaks with an implicated food, most resulted from food contaminated during preparation (92%) and food consumed raw (75%). Specific food categories were implicated in only 67 outbreaks; the most frequently named were vegetable row crops (e.g., leafy vegetables) (30%), fruits (21%), and mollusks (19%).

**Conclusions:**

Noroviruses are the leading cause of reported foodborne disease outbreaks and most often associated with contamination of food in restaurants during preparation by infected food workers.

**Implications for Public Health Practice:**

Improved adherence to appropriate hand hygiene, excluding ill staff members from working until ≥48 hours after symptom resolution, and supervision by certified kitchen managers are all recommended to reduce the incidence of foodborne norovirus disease.

## Introduction

Noroviruses are the leading cause of both sporadic cases and reported outbreaks of acute gastroenteritis (diarrhea or vomiting) in the United States (1,2). Each year, there are an estimated 19–21 million cases of norovirus disease, including 1.7–1.9 million outpatient visits, 400,000 emergency department visits, 56,000–71,000 hospitalizations, and 570–800 deaths, which result in approximately $777 million in health-care costs (2). Rates of severe outcomes, such as hospitalization and death, are greatest in children aged <5 years and older adults aged ≥65 years (2). Symptoms include vomiting, diarrhea, and sometimes fever, although norovirus infections also can be asymptomatic (3). This genetically-diverse group of viruses comprises six genogroups (GI–GVI), three of which (GI, GII, and GIV) cause human disease (4). Genogroups are further subdivided into at least 38 known norovirus genotypes; GII.4 strains cause most outbreaks worldwide (5).

Transmission of norovirus occurs primarily via the fecal-oral route, including direct person-to-person contact, consumption of contaminated food or water, or contact with contaminated environmental surfaces (3). Noroviruses also might be spread through incidental ingestion of vomitus droplets, which can disperse via aerosolization. The varied means through which noroviruses spread coupled with their environmental stability (remain infectious at freezing temperatures or until heated above 140°F [60°C], and for 2 weeks on surfaces), resistance to common disinfectants, low infectious dose (18–2,800 viral particles), and copious shedding (up to 10^12^ viral particles per gram of feces) among persons with asymptomatic infections as well as before, during, and after the manifestation of symptomatic infections make these viruses challenging to control (3,6,7).

Norovirus diagnostics generally rely on molecular methods and are not in widespread clinical use for sporadic cases; however, data collected through outbreak investigations provide insights that can help guide prevention efforts. Noroviruses often are associated with person-to-person spread in health-care settings, for which specific prevention and control guidelines are available* (8); however, noroviruses also are the leading cause of sporadic cases and outbreaks of foodborne disease in the United States (9,10), and thus require specific attention to improve food safety. This report provides an updated description of the epidemiology of U.S. norovirus outbreaks, focusing on those resulting primarily from foodborne transmission, to help target interventions.

## Methods

Since 2009, state, local, and territorial health departments have electronically reported data to CDC on outbreaks of acute gastroenteritis transmitted through food, water, person-to-person contact, animal contact, contaminated environments, and unknown transmission routes through the National Outbreak Reporting System† (NORS) (1). An outbreak was defined as two or more cases of a similar illness epidemiologically linked to a common exposure (e.g., a setting or a food). Primary transmission route is determined by each reporting site, based on the local public health investigation and CDC guidance documents.§ Outbreaks with a first illness onset date of January 2009–December 2012 that indicated norovirus as the only suspected or confirmed cause were included in this analysis.

Frequencies of norovirus outbreaks, outbreak-related illnesses, and their associated outcomes (i.e., outpatient visits, emergency department visits, hospitalizations, and deaths) were calculated. Demographic data were not always reported; therefore, the relative proportions of illnesses by age group and sex among those reports that included such data (47% and 65%, respectively) were extrapolated to the total number of reported outbreak-associated illnesses. Monthly counts of outbreaks stratified by primary transmission route were calculated to assess differences in seasonality. Rates of reported outbreaks were calculated by dividing the average annual number by the average U.S. intercensal population estimates from 2009–2012.¶ Proportions among categorical variables were compared using chi-square tests, and median illnesses per outbreak were compared by Wilcoxon rank-sum tests.

Level of food preparation (i.e., raw with minimal or no processing, raw with some processing, or cooked) and specific factors contributing to food contamination were analyzed using standardized categorization schemes.** Foods implicated in norovirus outbreaks were classified using a categorization scheme recently developed by the Interagency Food Safety Analytics Collaboration.††

## Results

**FIGURE 1:**
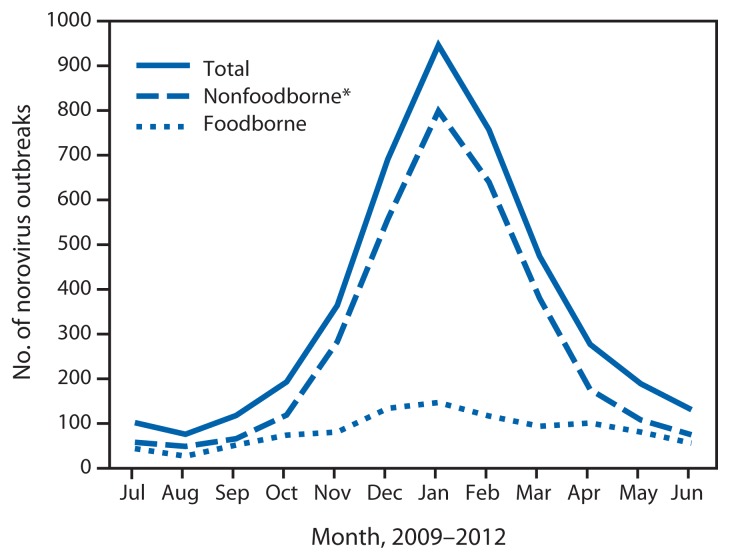
Number of reported norovirus outbreaks, by primary transmission mode and month of onset — National Outbreak Reporting System, United States, 2009–2012 * Includes person-to-person, waterborne, environmental contamination, and other or unknown transmission modes.

During 2009–2012, a total of 4,318 norovirus outbreaks were reported to NORS, resulting in 161,253 illnesses, 2,512 hospitalizations, and 304 deaths. Foodborne transmission was the primary mode reported in 1,008 (23%) norovirus outbreaks, representing 48% of the 2,098 foodborne outbreaks reported with a single suspected or confirmed cause during the 4-year study period. Other primary transmission modes reported among the 4,318 norovirus outbreaks included person-to-person (2,976 [69%]), environmental (15 [0.35%]), waterborne (11 [0.26%]), and unknown transmission mode (308 [7%]). In 158 (16%) of foodborne norovirus outbreaks, secondary transmission through one of these other modes was reported. Norovirus outbreaks were most common in winter, with 2,394 (55%) occurring during December–February (Figure 1). Among foodborne norovirus outbreaks, 398 (39%) occurred during December–February, compared with 1,996 (60%) of nonfoodborne norovirus outbreaks.

Of the 4,318 reported norovirus outbreaks, 2,961 (69%) were laboratory-confirmed, and 1,357 (31%) were suspected to be caused by norovirus based on clinical or epidemiologic findings. Of confirmed norovirus outbreaks, a genogroup was identified in 2,729 (92%), including 2,341 (86%) GII, 374 (14%) GI, 13 (0.5%) mixed GI/GII, and one (0.04%) GIV. A specific norovirus genotype was reported in 707 (24%) of the laboratory-confirmed outbreaks, among which GII.4 (465 [66%]) was predominant, followed by GII.1 (58 [8%]) and GI.6 (56 [8%]). Foodborne outbreaks were more often caused by non-GII.4 genotypes (48%) than were nonfoodborne outbreaks (31%, p<0.001).

**FIGURE 2:**
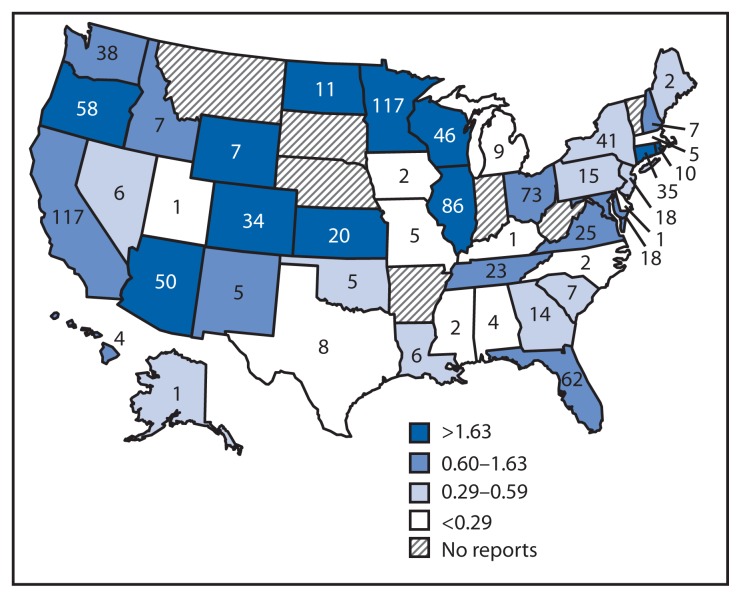
Number and rate of reported foodborne norovirus outbreaks (per 1 million person-years*), by state — National Outbreak Reporting System, United States, 2009–2012 * Legend indicates rate ranges divided by quartile.

Foodborne norovirus outbreaks were reported by 43 states (Figure 2), with the number per state ranging from one to 117 (median = nine). The median number of outbreaks per 1,000,000 person-years reported among the states was 0.6 (range = 0.05–5.5). Of 1,008 foodborne norovirus outbreaks, a setting of food preparation was reported for 904 (90%), among which restaurants (574 [64%]) and catering or banquet facilities (151 [17%]) were most common (Table 1). In contrast, most (80%) nonfoodborne outbreaks occurred in long-term care facilities such as nursing homes.

Demographic characteristics and outcomes of outbreak-associated illnesses reflected the settings in which outbreaks occurred (Table 2). Foodborne outbreaks more often affected men (44%) and persons aged <75 years (95%), compared with nonfoodborne outbreaks (30% men and 50% aged <75 years, both p<0.001). Likewise, the reported case-hospitalization and case-fatality ratios in foodborne outbreaks (1% and 0.01%, respectively) were lower than those in nonfoodborne outbreaks (2% and 0.3%, respectively, both p<0.001), However, a greater proportion of cases among foodborne outbreaks resulted in emergency department visits than among nonfoodborne outbreaks (4% versus 2%, p<0.001). Foodborne outbreaks also had significantly fewer reported cases (median: 12 per outbreak) compared with nonfoodborne outbreaks (median: 30 per outbreak, p<0.001).

Factors contributing to food contamination were reported in 520 (52%) of 1,008 foodborne norovirus outbreaks, among which infectious food workers were implicated as the source of contamination in 364 (70%). Bare-hand contact with ready-to-eat foods was explicitly identified in 196 (54%) of these outbreaks.

At least one specific food item was implicated in 324 (32%) of 1,008 foodborne norovirus outbreaks; among those outbreaks with data, 92% of implicated foods were contaminated during preparation, and 75% were foods eaten raw (i.e., not cooked). Of 324 outbreaks with an implicated food, only 67 (21%) could be attributed to a single food category; those attributed most often were vegetable row crops (e.g., lettuce and other leafy vegetables) (20 [30%]), fruits (15 [21%]), and mollusks (13 [19%]).

## Conclusions and Comment

This report highlights the predominant role of noroviruses among foodborne disease outbreaks and specific actions that might reduce their impact on public health. While there is the potential for norovirus contamination during production or harvesting of foods commonly eaten raw, particularly molluscan shellfish and fresh produce (10), most norovirus contamination occurs during food preparation. As shown in a previous analysis of foodborne norovirus outbreaks occurring during 2001–2008 (10), food workers continue to be the primary source of contamination and have the potential to significantly amplify community transmission of noroviruses through widespread exposure. The majority of reported foodborne norovirus outbreaks result from foods prepared in restaurants and other food service settings, where bare-hand contact by infectious workers with ready-to-eat foods frequently is identified. Thus, interventions targeting food workers have substantial potential for prevention of norovirus transmission.

Steps to curtail contamination of ready-to-eat foods by food workers include 1) adherence to appropriate recommendations for hand washing and avoiding bare-hand contact with ready-to-eat foods (e.g., through use of gloves or utensils), 2) compliance with policies to prevent ill staff members from working until ≥48 hours after symptom resolution, 3) and supervision by a certified kitchen manager, as recommended by the Food and Drug Administration Food Code (11). However, an observational study of food workers in restaurants found proper hand washing in only 27% of activities for which it is recommended and even less frequently (16%) when gloves were used (12). Additionally, one in five food workers in restaurants report having worked while ill with vomiting or diarrhea for at least one shift in the previous year (13). Fear of job loss and concerns about leaving coworkers short-staffed were identified as significant factors in their decision to work while ill and thus are important barriers to be addressed (13). One specific intervention with demonstrated success is the training and certification of kitchen managers in appropriate food safety practices; supervision by such certified kitchen managers is associated with fewer norovirus outbreaks and absence of bare-hand contact with ready-to-eat foods as a contributing factor when outbreaks do occur (14).

The findings in this report are subject to at least three limitations. First, the 100-fold difference in outbreak reporting rates between the highest and lowest reporting states and the fact that some states did not report any outbreaks likely reflect differing sensitivities of surveillance for ascertaining outbreaks, rather than variation in disease incidence alone. The actual incidence likely is much higher, indicating a continued need for capacity of state and local health departments to investigate and report outbreaks. Second, missing data in key NORS report fields, such as “contributing factors” and “implicated foods,” indicate the need for investigative resources to understand the causes of an outbreak (15). CDC efforts to address these issues include improved integration of NORS with other surveillance systems, such as the National Voluntary Environmental Assessment Information System§§ and CaliciNet¶¶ (4), the CDC-coordinated laboratory network for norovirus outbreaks. Data from these systems in conjunction with NORS data might help improve attribution of norovirus disease to specific strains and environmental contributing factors. Additionally, the Norovirus Sentinel Testing and Tracking*** network can help improve data completeness and provide a real-time assessment of norovirus activity in the context of new strain emergence (16). Finally, NORS does not capture outbreaks occurring on cruise ships with international and U.S. ports; those are reported through an active surveillance collaboration between the cruise industry and the CDC Vessel Sanitation Program.††† Although the 44 norovirus outbreaks meeting Vessel Sanitation Program posting criteria during 2009–2012 would represent only 1% of those reported through NORS, these high-profile outbreaks often result in large numbers of cases.

The public health burden exacted by noroviruses is substantial. Although candidate norovirus vaccines are in development and show promise (17), behavioral interventions focused on food workers continue to be primary means to prevent foodborne norovirus disease. Provisions in the Food and Drug Administration Food Code (11) outline how foodborne spread of noroviruses can be curtailed and food safety improved.

**Key Points**

- Norovirus is highly contagious and can cause severe disease. About 1 in 15 U.S. residents become ill with it each year and up to 800 die.
- Person-to-person contact and foodborne transmission are the main ways that norovirus outbreaks occur.
- Of those foodborne outbreaks in which a single cause was identified, 48% were caused by norovirus, making it the leading cause of foodborne outbreaks in the U.S.
- Restaurants are the most common setting for foodborne norovirus outbreaks. Food handlers infected with norovirus are the largest source of food contamination.
- CDC recommends improved adherence to hand hygiene, having ill workers stay home until ≥48 hrs after their symptoms resolve, and having kitchen managers become formally certified in food safety.
- Additional information is available at http://www.cdc.gov/vitalsigns.

## Figures and Tables

**TABLE 1:** Number and percentage of reported foodborne and nonfoodborne norovirus outbreaks, by setting* — National Outbreak Reporting System, United States, 2009–2012

	Foodborne		Nonfoodborne†	
		
Setting	No.	(%)	No.	(%)
Restaurant	574	(64)	38	(1)
Catering or banquet facility	151	(17)	8	(0.3)
Private residence	37	(4)	32	(0.1)
School	13	(1)	148	(6)
Long-term care facility	12	(1)	2,060	(80)
Hospital	2	(0.2)	115	(4)
Day care	1	(0.1)	52	(2)
Other/Multiple settings	114	(13)	137	(5)
All settings	904	(100)	2,590	(100)

* A setting was reported in 904 (90%) of 1,008 foodborne outbreaks and in 2,590 (78%) of 3,310 nonfoodborne outbreaks.

† Includes person-to-person, waterborne, environmental contamination, and other or unknown transmission modes.

**TABLE 2:** Number and percentage of persons with illness associated with reported foodborne and nonfoodborne norovirus outbreaks, by selected characteristics and outcomes — National Outbreak Reporting System, United States, 2009–2012

	Foodborne		Nonfoodborne*	
		
Characteristic/Outcome	No.	(%)	No.	(%)
**Sex** †				
Male	9,285	(44)	42,112	(30)
Female	11,780	(56)	98,076	(70)
**Age group (yrs)** †				
0–4	481	(2)	2,178	(2)
5–19	2,959	(14)	18,621	(13)
20–49	9,558	(45)	24,619	(18)
50–74	7,002	(33)	24,910	(18)
≥75	1,064	(5)	69,860	(50)
**Outcomes** §				
Outpatient visit	1,102	(7)	3,848	(7)
Emergency department visit	520	(4)	1,109	(2)
Hospitalization	203	(1)	2,309	(2)
Death	2	(0.01)	302	(0.3)
**Total illnesses**	**21,065**	**(100)**	**140,188**	**(100)**

* Includes person-to-person, waterborne, environmental contamination, and other or unknown transmission modes.

† Proportions of illness by age and sex among persons for whom such data were reported were extrapolated to include all patients from reported norovirus outbreaks, including those without such data.

§ Proportions based on specific known outcomes where such data were reported; thus, each proportion was calculated using a different denominator.

## Footnotes

* Additional information available at http://www.cdc.gov/norovirus/php/responding.html.

† Additional information available at http://www.cdc.gov/nors.

§ Available at http://www.cdc.gov/nors/pdf/nors_guidance_20130219_508c.pdf.

¶ Additional information available at http://www.cdc.gov/nchs/nvss/bridged_race.htm.

** Available at http://www.cdc.gov/nors/pdf/nors_appendix_v3.pdf.

†† Additional information available at http://www.cdc.gov/foodborneburden/pdfs/ifsac-webinar-06-18-2013-slides-508c.pdf.

§§ Additional information available at http://www.cdc.gov/nceh/ehs/nveais/index.htm.

¶¶ Additional information available at http://www.cdc.gov/norovirus/reporting/calicinet.

*** Additional information available at http://www.cdc.gov/norovirus/reporting/norostat.

††† Additional information available at http://www.cdc.gov/nceh/vsp/surv/gilist.htm.
